# Endocarditis after Use of Tongue Scraper

**DOI:** 10.3201/eid1309.070544

**Published:** 2007-09

**Authors:** Andrew M. Redmond, Cathryn Meiklejohn, Timothy J. Kidd, Robert Horvath, Christopher Coulter

**Affiliations:** * The Prince Charles Hospital, Chermside, Queensland, Australia; † Pathology Queensland, Herston, Queensland, Australia

**Keywords:** Endocarditis, tongue scraping, Haemophilus parainfluenzae, gram negative, HACEK, letter

**To the Editor:** Tongue scraping is advocated as a therapy for managing halitosis and as a technique for preventing dental caries by reducing bacterial counts in the mouth (*1*). The practice has been in existence for centuries (*2*). A Cochrane review has concluded that tongue cleaning is marginally and temporarily more effective than use of a toothbrush in reducing a measurable marker for halitosis, exhaled volatile sulfur compounds (*3*). The use of tongue scrapers may not be limited to those with clinical halitosis, as 10%–30% of Americans report bad breath (*4*), and websites offer to solve the problem of “your bad breath” for a price. We report the case of a woman in whom infective endocarditis followed the use of a tongue scraper.

A 59-year-old woman with a known history of mitral valve prolapse with associated valvular regurgitation had onset of progressive malaise, fever, sweats, myalgia, and headache; the symptoms lasted 10 days. Two months previously she had begun cleaning her tongue with a plastic tongue scraper purchased at her local pharmacy. She had not undergone recent dental work. Her medical background included migraines, hypertension, mild quiescent psoriasis, and previous depression. Her medications were venlafaxine and candesartan.

When seen at her local hospital, she reported severe headache and myalgia, with fever. The same day, she had a rigor at home and reported chest tightness and mild dyspnea. Physical examination showed no focal findings other than the mitral valve prolapse. A provisional diagnosis of bacterial meningitis was made. Emergency treatment comprised intravenous dexamethasone, ceftriaxone, and benzylpenicillin. Cerebrospinal fluid analysis performed shortly after showed no cells and normal glucose and protein levels. Culture of the cerebrospinal fluid was negative. No further antimicrobial agents were administered. Multiple blood cultures were drawn but remained culture negative. Serologic tests for Q fever, *Bartonella* spp., and endemic rickettsiae were negative. She continued to be febrile.

A transthoracic echocardiogram showed dilatation of the mitral valve annulus with bi-leaflet prolapse and vegetation attached to the anterolateral commissure. She was referred to a tertiary care center, and therapy with penicillin, flucloxacillin, and gentamicin was begun for culture-negative endocarditis. Transesophageal echocardiography and visual examination at the time of valve replacement confirmed the presence of large valvular vegetations. After infected tissue was excised, a prosthetic mitral valve was placed. Extended culturing of the blood failed to identify a pathogen. Histopathologic examination of the explanted valve identified fibrinopurulent vegetations with destruction of the valve leaflet. The excised material was split into sections and submitted for culture; all demonstrated a scant growth of *Haemophilus parainfluenzae*. This finding was identified by a Remel RapID NH Panel (Remel, Lenexa, KS, USA) and confirmed by 16S rRNA gene sequencing. The patient was treated with ampicillin and gentamicin for 2 weeks. She then had 4 further weeks of therapy with daily ceftriaxone at home. She is now well.

This patient’s endocarditis was most likely caused by bacteremia from tongue scraping, and the abnormal valve is likely to have been a predisposing factor. The link between oral flora and endocarditis has long been recognized (*5*), and guidelines for prophylactic use of antimicrobial agents before dental manipulation are established. A literature review did not show any previous reports of endocartitis associated with use of a tongue scraper. There are numerous reports of endocarditis after tongue piercing, with a variety of organisms including viridans streptococci, *H. aphrophilus, Neisseria mucosa*, and methicillin-resistant *Staphylococcus aureus* (*6*–*9*). Most of these articles reported a pre-existing valvular abnormality, as in our case. Bacteremia caused by routine tooth brushing does not appear to be clinically important, and there are conflicting data about its frequency (*10*). The inoculum of bacteria transmitted into the bloodstream with brushing may be smaller than that with tooth extraction. Given the frequency of routine tooth brushing, antimicrobial prophylaxis is impractical in any case. The practice of tongue scraping, however, has not been well studied, and both the magnitude and frequency of bacteremia may be greater than with routine tooth brushing.

We propose that our patient’s infective endocarditis was most likely a consequence of bacteremia from her use of a tongue scraper. Persons with abnormal cardiac valves and intravascular devices such as pacemakers may be at particular risk. Patients with previous infective endocarditis and high-risk cardiac valve defects should be informed that tongue scraper use is not prudent.
